# Direct Human Contact with Siloxanes (Silicones) – Safety or Risk Part 1. Characteristics of Siloxanes (Silicones)

**DOI:** 10.3389/fphar.2016.00132

**Published:** 2016-05-30

**Authors:** Krystyna Mojsiewicz-Pieńkowska, Marzena Jamrógiewicz, Katarzyna Szymkowska, Dominika Krenczkowska

**Affiliations:** Department of Physical Chemistry, Faculty of Pharmacy with Subfaculty of Laboratory Medicine, Medical University of GdańskGdańsk, Poland

**Keywords:** siloxanes, methylsiloxanes, low molecular siloxanes, silicones, physical properties of siloxanes, chemical properties of siloxanes, safety of using silicones, risk of using silicones

## Abstract

Siloxanes are commonly known as silicones. They belong to the organosilicon compounds and are exclusively obtained by synthesis. Their chemical structure determines a range of physicochemical properties which were recognized as unique. Due to the susceptibility to chemical modifications, ability to create short, long or complex polymer particles, siloxanes found an application in many areas of human life. Siloxanes differ in particle size, molecular weight, shape and chemical groups. As a result, this determines the different physico-chemical properties, that directly affect the safety or the risk of their use. The areas that can be a source of danger to human health will be commented in this paper.

## Introduction

Siloxanes (silicones) constitute a group of low molecular weight compounds, organosilicon oligomers and polymers, which play a very important role in human life. The dynamic development of silicones technology can be observed last years, which caused that more than 150,000 of practical applications have been registered, including pharmaceutical, medical, cosmetic, and food production (CES – Silicones Europe, 2008; Chemical Economics Handbook, 2013; Tran et al., 2015; Xu et al., 2015). It should be emphasized that the global capacity of siloxanes production in 2002 amounted to 2,000,000 tons, and currently it is more than 8,000,000 tons (Tran et al., 2015). Since 2002 **a**nnual average production amount of only cyclic methylsiloxanes was 470,000 tons in the US, and 800,000 tons in China (Tran et al., 2015; Xu et al., 2015). The largest consumption of silicones was noted in China, Western Europe and North America. It is estimated that the global market for siloxanes (silicones) will reach more than 19 billion dollars by the year 2017 (Chemical Economics Handbook, 2013; Frust, 2014; Mojsiewicz-Pieńkowska, 2015). These indices suggest that this is a very serious branch of the economy in many countries. Siloxanes are important object of the research due to their widespread use not only by adults and children, but also by infants. Currently, nearly 50% of new skin care products contain at least one type of silicone (Schalau and Ulman, 2009). Due to the fact that siloxanes differ in particles size, molecular weight, shape and physico-chemical properties, the discussion concerning their direct effect on safety or risk of an application was undertaken in this study.

## Characteristics of Siloxanes (Silicones)

### The Beginnings of Silicones Chemistry and Contemporary Manufacturers

Thousands of scientific articles and patents have been created since 1872, when the first siloxane polymer was obtained. A rapid development of the synthesis of organosilicon compounds was noted in subsequent years. Charles Friedel and James Crafts obtained tetraethylsilane, which was the first compound containing Si-C bond (Brook, 2000; Butts et al., 2002). However, the greatest achievements in the field of synthesis are attributed to Frederic Stanley Kipping, who in 1904 used the Grignard reaction for the preparation of organosilicon compounds, and discovered the intermolecular condensation of silanols leading to obtain siloxanes and polysiloxanes. Rapid development of silicones chemistry occurred in the thirties of the 20th century. Technological breakthrough was the development of so called direct methylchlorosilanes synthesis method by Eugene Rochow in the United States in 1941 (Brook, 2000; Chojnowski and Cypryk, 2000; Butts et al., 2002; Thomas, 2010). Methylchlorosilanes became the starting compounds (monomers) in various preparation reactions of silicones. The technology for direct synthesis was developed nearly at the same time, in the year 1942 in Germany, under the supervision of Richard Miller (Butts et al., 2002). The production of silicones, the first generation of these unique polymers, started during World War II in the USA in Dow Corning Corporation (Chojnowski and Cypryk, 2000; Thomas, 2010). Currently, the major leaders in silicones production include the following companies: Wacker Chemie (Germany), Dow Corning (USA), SiVance (USA), Shin-Etsu Chemical (Japan), NuSil Technology (USA), Momentive Performance Materials (USA), Evonik Industries (Germany), Qingdao Ocean Chemical Co, Ltd (China), Bluestar Silicones (International Corporation), Mizusawa Industrial Chemicals Ltd (Japan).

### Chemical Structure and Particle Geometry

Chemical structure of the compound, as well as particle size, should be taken into account in safety evaluation of the use of siloxanes. These two factors determine the physico-chemical properties, which strictly correlate with the ability to interact with the human organism, as well as the ability to pass through cellular barriers.

**FIGURE 1:**
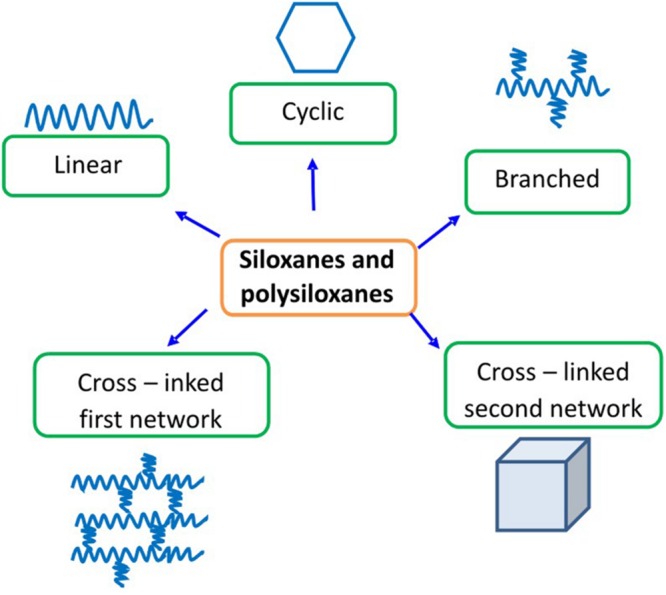
**Five basic structures of siloxanes**.

Silicon (Si) as a chemical element is more electropositive than carbon (C) and, like carbon, forms covalent bonds with different elements, e.g., with oxygen (O) (siloxanes and polysiloxanes), nitrogen (N) (silazanes and polysilazanes), sulfur (S) (siltianes and polysiltianes), silicon (Si) (silanes and polysilanes), carbon (C) (carbosilanes and polycarbosilanes) (Brook, 2000; Butts et al., 2002). This is an element that forms a wide variety of group of organosilicon compounds. Undoubtedly, the most crucial group of organosilicon compounds are siloxanes, which are commonly known as silicones. The name “silicones” originates from a time when it was thought that siloxanes have a structure analogous to the ketones Si = O. The studies proved that silicon does not have the ability to create stable double bonds. The correct structure of siloxanes is described by the repeating group —[R_2_Si–O]— with usually organic substituents at the silicon atom. Substituent R may usually represent the groups such as: methyl, ethyl, propyl, phenyl, fluoroalkyl, aminoalkyl, hydroxy, mercapto, hydrogen, vinyl. The chemistry of siloxanes distinguishes four levels of functionality, which, together with the type of functional groups, determine structure geometry. These are the monofunctional units (M) which act as terminators of the chains, difunctional ones (D) which form the multiparticle skeleton of oligomers and polymers linear chains, or cyclic compounds, trifunctional (T) and tetrafunctional (Q), which result in branched and spatially cross-linked molecules, observed in resins, rubbers and elastomers (Tomanek, 1991; Fendinger et al., 1997; Kuo, 1999; Dow Corning, 2000–2016c; Butts et al., 2002; Colas and Curtis, 2004; Omidian et al., 2011; Mojsiewicz-Pieńkowska, 2012, 2014). Siloxane molecule may be thus linear, cyclic, branched or cross-linked (**Figure 1**) (Dow Corning, 2000–2016a; Mojsiewicz-Pieńkowska, 2015). The characteristic of these principal structures are presented in **Table 1** (Chojnowski and Cypryk, 2000; Dow Corning, 2000–2016a; Mojsiewicz-Pieńkowska, 2015).

**Table 1:** The characteristic of principal structures of siloxanes.

No.	Structure	Characteristic
1	Linear	Volatile (e.g., dodecamethylpentasiloxane in cosmetics) and non-volatile liquids (e.g., dimethicone in oral capsules)
2	Cyclic	Volatile liquids (e.g., cyclomethicone in hairspray or creams)
3	Branched	Resins and rubbers, e.g., Soft Skin Adhesives from Dow-Corning company DC 7-9800 Part A, DC 7-9800 Part B in silicone tapes on the skin, or in transdermal systems;
4	Cross-linked (first network)	Resins, rubbers, elastomers (e.g., Cica-Care for the treatment of scars and keloids);
5	Cross-linked (second network)	Resins readily transformed into a three-dimensional network)

#### Comment

In conclusion, it should be noted that the safety of application decreases with the decrease in silicone particle size. This is due to the possibility to overcome biological membranes and skin barriers by low molecular siloxanes, which demonstrate a lipophilic character according to the rule of Lipinski. The bioavailability and also permeation of compounds through the skin layers is possible, when they meet the following conditions (Lipinski, 2000; Lipinski et al., 2001; Mojsiewicz-Pieńkowska, 2014):

(1) there are less than 5 hydrogen-bond donors (Expressed as the sum of hydroxide groups OHs and amine groups NHs);
(2) molecular Weight is less than 500;
(3) the Log P is less than 5;
(4) there are less than 10 hydrogen-bond acceptors (expressed as the sum of nitrogens and oxygens).

Therefore, consideration of siloxanes toxicity should be always referred to a particular compound, which may be low molecular, medium molecular (oligomer) or a high molecular weight (polymer), but not to the entire chemical group. Actually, particle size and chemical structure determine their physicochemical properties (e.g., solubility, lipophilicity, log P, volatility), ability to penetrate and permeate through skin layers, ability to overcome cellular barriers and toxicity. Moreover, low molecular weight silicones can change the structure of lipid bilayer, by the fluidization or even extraction of the lipids (Glamowska et al., 2015; Mojsiewicz-Pieńkowska et al., 2015; Yang and Guy, 2015). This effect can weaken the natural barrier of cell membranes (Glamowska et al., 2015; Mojsiewicz-Pieńkowska et al., 2015). Low molecular weight silicones can accumulate in the organism, and affect the organs in the long-term perspective (Wang et al., 2013). The least safe are cyclic siloxanes and low molecular weight linear siloxanes (Flassbeck et al., 2001; Gaubitz et al., 2002; Papp et al., 2004; Tran et al., 2015; Xu et al., 2015). Additionally, their volatility poses a threat of entering to the organism through the respiratory system (Genualdi et al., 2011; Kierkegaard and McLachlan, 2013; Tran et al., 2015; Xu et al., 2015).

### Synthesis of Linear and Cyclic Siloxanes and Polysiloxanes

The methods of siloxanes synthesis, and the possibility of purification from reagents or intermediates, should be taken into consideration in these compounds safety evaluation.

Siloxanes are generally obtained from monomers, such as: methyltrichlorosilane, dimethyldichlorosilane, methylphenyldich-lorosilane, diphenyldichlorosilane, or phenyltrichlorosilane (Brook, 2000; Butts et al., 2002). In the chemical industry, they are obtained using the three methods: Grignard method, direct method using silicon reaction with an alkyl chloride (so-called Rochow process using a copper catalyst) or using an addition method (Brook, 2000; Clarson, 2003; Colas and Curtis, 2004; Daum, 2005). The siloxanes are prepared in a few stage process. Reactive monomers of chlorosilanes obtained using one of the three mentioned methods are subjected to hydrolysis with an excess water at a temperature range of 10–90°C in organic solvents, e.g., esters and ketones. Continuous hydrolysis of, e.g., dimethyldichlorosilane (Me_2_SiCl_2_) allows to obtain a mixture of cyclic and linear hydroxyl-terminated oligosiloxanes. The hydrolyzate is then processed in linear dimethylsiloxane polymers in one of two industrial processes: polymerization or condensation. Polymerization process results in an opening of cyclic siloxanes ring, after the use of cracking and H^+^ or OH^-^ catalyst. It should be noted that the hydrolyzate in the cracking process in the presence of a strong base is first converted to a mixture of cyclic monomers, especially octamethylcyclotetrasiloxane (D4) and decamethylcyclopentasiloxane (D5), and ring opening occurs after that. At the end of the reaction, the reactor contains the mixture of linear polysiloxane and about 15% of cyclic oligomers (Noll, 1968; Voronkov et al., 1978; Rubinsztajn et al., 1989; Colas and Curtis, 2004). Polymerization of cyclic siloxanes was elaborated in the US in the fifties by General Electric Co. and Dow Corning (Noll, 1968; Rich et al., 1997). The polycondensation of linear hydroxyl-terminated oligosiloxanes using a catalyst of linear phosphonitrilic chloride produces linear high molecular polymers. This process was developed in Germany and was used on an industrial scale, e.g., in Wacker Chemie AG (Nitzsche and Wick, 1958; Nitzsche et al., 1965). The polycondensation also produces a mixture of linear polymers and cyclic oligomers, but with a low content of about 2% (Nitzsche et al., 1974).

#### Comment

**FIGURE 2:**
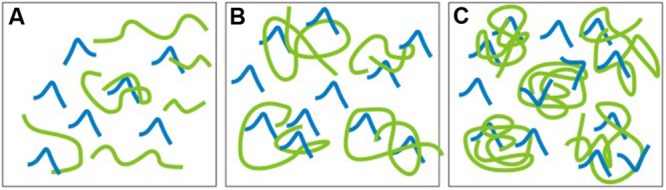
**Scheme of the possibility of siloxanes short chains trapping in the bundles of longer chains: (A)** Viscosity <10 000 cSt, small chains entanglement; **(B)** Viscosity 60 000 cSt, moderate chains entanglement, **(C)** Viscosity 30 000 000 cSt high chains entanglement.

In conclusion, considering the safety of the direct application or contact by humans with siloxanes, the polycondensation reaction is preferred, due to lower contamination with low molecular weight siloxanes of cyclic structure. The literature indicates that they exhibit toxic effects, for example: cancerogenicity, modifications in proteins conformation, influence on the immune system, genotoxicity, skin irritations, intraocular pressure increase and teratogenicity (Wang et al., 2013; Mojsiewicz-Pieńkowska, 2014). Also the presence of low molecular weight siloxanes with linear structure that are formed during the course of synthesis stages, but are not efficiently removed during the purification of oligomers and polymers, e.g., by distillation, should be monitored. Moreover, except from inefficient distillation, there is also another source of contaminants. Longer chains can form, for example, so called “traps” for low molecular weight compounds (with short chains), impeding the distillation of both linear, and cyclic siloxanes (Dow Corning). **Figure 2** presents the scheme of a possibility of siloxanes short chains trapping in the bundles of long chains (Dow Corning, 2000–2016b). This is possible due to the fact, that short and long chains are chemically compatible with each other.

Due to the specificity of siloxanes synthesis reaction, which is the cause of formation of the chains with different numbers of structural units in the molecule, the obtained product should be treated not as a specific compound, but as a mixture of polymers, oligomers, and even low molecular weight compounds of different chain lengths and thus different molecular weights. Although there is a predominant fraction of a specific degree of polymerization in each sample after the synthesis, also referred to as the dominant fraction, there is the possibility of low molecular contamination occurrence. The mixture may also include the siloxanes of cyclic structure (Mojsiewicz-Pieńkowska, 2014).

It should also be noted that human can have an undesired contact with the low molecular weight siloxanes of the linear or cyclic structure, not only in the form of contaminations. The problem may result from a deliberate action of the manufacturers to obtain the product with desired viscosity. The possibility of trapping was used in industry in order to obtain specific viscosity as a result of mixing of polymers with different degrees of polymerization. There are even special diagrams facilitating the selection of appropriate quantities of polysiloxanes for the mixing (Catalog Gelest, 2012). Therefore, the viscosity parameter, determining the physical characteristic of particular analyte, is not sufficient to determine whether it deals, for example, with active pharmaceutical ingredient (Dimeticone, Simeticone) or a food additive (E-900) in accordance with the standard (Mojsiewicz-Pieńkowska and Łukasiak, 2003; Mojsiewicz-Pieńkowska, 2008, 2011, 2012, 2014).

### Synthesis of Silicone Elastomers

The synthesis of the silicone elastomers involves the process of vulcanization as a result of cross-linking the linear siloxane polymers, so called reactive polymers. The cross-linking process involves conversion of linear polymer molecules in the spatial macromolecule, which is the consequence of cross-links, so called bridges (nodes), formation between them. Parts A and B of the individual components contribute in the chemical reactions leading to rubber cross-linking. The cross-linker is a multifunctional polysiloxane with hydrosilane substituent Si-H, and the second one is the basic siloxane polymer with vinyl groups (CH = CH_2_), containing Karstedt’s catalyst Pt_2_{[(CH_2_ = CH)Me2Si]_2_O}_3_. The catalyst is a chemical compound containing two platinum atoms, which attach two divinyltetramethyldisiloxyl ligands, and one tetramethyldisiloxyl addend connects both platinum atoms. The mixing of two components A and B is followed by the quick addition reaction, so called hydrosilylation. It involves the attachment of one molecule to another which results in only one product, with no by-products. The addition reaction takes place due to disruption of multiple carbon–carbon bond of the vinyl moiety, and results in silicon-carbon bond formation. Polyaddition reaction of Si-H bonds to vinyl groups results in the formation of numerous hydrocarbon bridges connecting the polysiloxane chains. This is the type of cross-linking with their ends participation (endlinking) (Mojsiewicz-Pieńkowska et al., 2011, 2015).

#### Comment

**FIGURE 3:**
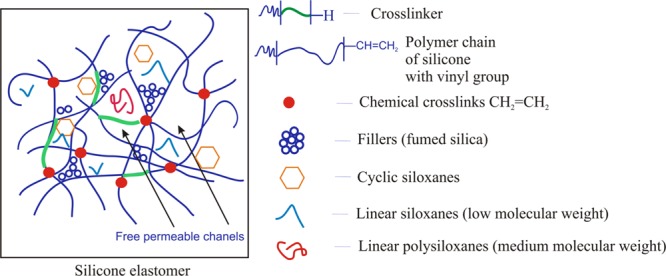
**Scheme of short chain siloxanes with a linear and cyclic structure closing in the elastomer network**.

Having in mind the safety of direct application or contact by humans with the siloxane (silicone) elastomers, it is necessary to use reactive linear polymers free of contamination with cyclic siloxanes, as well as low molecular polysiloxanes with linear structure. When the cross-linking reaction involves rubbers contaminated with cyclic siloxanes or low molecular linear siloxanes, they are enclosed in the network. **Figure 3** presents the scheme of closing of the short chain of siloxanes with a linear, as well as cyclic structure, in elastomer’s network. This is possible due to the fact that the elastomer has got some spaces between cross-linked chains, so-called free permeable channels. For example, in the contact with skin, the short chains of polysiloxanes with a linear and cyclic structure can diffuse from the elastomer into the *stratum corneum* and deeper layers of the skin (Sanchez et al., 2003; Mojsiewicz-Pieńkowska et al., 2015).

### Fundamental Siloxanes Characteristic Related to Chemical Structure

**FIGURE 4:**
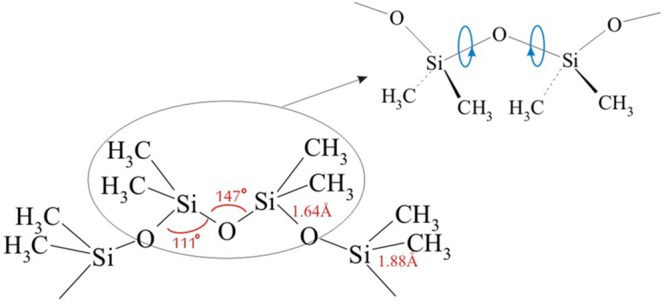
**Example of siloxane chain**.

Chemical structure of siloxanes caused that they are distinguished by many features that allowed their dynamic development, diversity and wide application. Especially significant is an unusual static flexibility of siloxane chain manifested by a huge number of available conformations, as well as dynamic flexibility related to the ease of rapid conformational changes (Tiwari and Soucek, 2014). The extraordinary flexibility of polysiloxanes chain results from relatively long Si-O (about 1.64 Å) and Si-C bonds (about 1.88 Å), and the lack of substituents at every second atom of the chain. The flexibility of the chain facilitates rotation around chemical bonds (**Figure 4**). The alternating location of Si and O atoms results in a specific arrangement of electron interactions. Si-O bond also has a high proportion of ionic character (about 50%), which causes higher strength of binding compared to the equivalent of binding with C (De Buyl, 2001). The consequences of mentioned effects are very low energy barrier of Si-O-Si angle (<0.5 kcal/mol) and rotation around Si-O bond. This results in a very fast relaxation of conformation stress, leading to adaptation of chain conformation to ambient conditions (Salamone, 1996; De Buyl, 2001; Luria, 2003; Lin et al., 2009; Pinto et al., 2010; Mojsiewicz-Pieńkowska et al., 2015).

**FIGURE 5:**
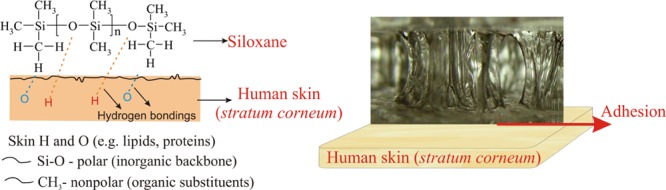
**Ability to form hydrogen bonds with human skin as a result of the amphiphilic nature of siloxanes and adhesion of silicone elastomer to the skin**.

Siloxanes are also characterized by an amphiphilic character (**Figure 5**). On the one side, there is an inorganic chain with strong polar Si-O bonds, and on the other side non-polar organic groups bonded to silicon atom (Mojsiewicz-Pieńkowska, 2015; Physical and chemical properties of silicone). The amphiphilic nature precisely plays an essential role in their unique properties. An example can be the ability to form hydrogen bonds with human skin, by contacting an inorganic part containing oxygen atoms (Si-O), which act as hydrogen acceptors, or an organic part (CH_3_), which represent hydrogen donors. This feature determines the phenomenon of adhesion between the siloxane elastomer and human skin (Mojsiewicz-Pieńkowska et al., 2011, 2015).

### Siloxanes Characteristics Relevant for Medical and Cosmetic Applications

The chemical structure is related to the characteristic features of the siloxanes and polysiloxanes, which determine the beneficial and unique properties. As a result, they are particularly used in many areas of human life.

The specific characteristics of siloxanes which determine their beneficial properties include strong Si-C and Si-O bonds, free rotation of the chain, and partially ionic nature of the siloxane bond. An ability of free rotation of the chain enables an optimal orientation of, for example, alkyl groups on the surface of oligomer or polymer. Hence, in contact with an environment, the surface of the layer of low energy (e.g., dimethicone polydimethylsiloxane) is dominated by densely packed methyl groups, which causes low energy and chemically inert surface. The chemical inertness (lack of reactivity) is also affected by strong Si-C and Si-O bonds (De Buyl, 2001; Butts et al., 2002; Mojsiewicz-Pieńkowska, 2011, 2014, 2015). This is particularly important for the safety of siloxanes and polysiloxanes application in a direct contact with human organism. In turn, ionic nature causes higher susceptibility to heterolytic rupture, which contributes to the formation of cation and anion, not radicals as in case of homolytic rupture (De Buyl, 2001). This feature enhances the safety of siloxanes application in contact with human organism.

Due to their dual nature (amphiphilic character), siloxanes are also characterized by a quick diffusion at the interface, where they obtain the conformation corresponding to the minimum free energy of the surface, which makes it possible to create a thin, highly adherent layer on it. Amphiphilic character (**Figure 5**) affects thus a range of surface properties of siloxanes, which results in a very broad applicability of these compounds. This may cause a decrease in surface tension (e.g., oral activity of dimethicone) or adhesion (e.g., Silicone Soft Skin Adhesive -SSA elastomer) effect on the skin. When elastomers are in contact with an air, then the pendant methyl groups are compressed by the contact surface. This is an important phenomenon, since methyl group determine the hydrophobic properties. In turn, in case of the contact with water, the dipole of siloxane skeleton is a predominant part, responsible for interactions between the media. This causes that the surface of siloxane (silicone) elastomer becomes more hydrophilic (Kurian et al., 2003). This aspect is extremely essential in the understanding of the differences in adhesion properties between silicone elastomers. Moreover, trends of Si-O siloxane chain to a slight elongation under a small force at a room or higher temperature, and recovery to its original form after deflecting force elimination, optimize the adhesion strength and allow, e.g., using silicone film on the skin for a few hours (De Buyl, 2001; Mojsiewicz-Pieńkowska, 2011, 2014, 2015).

Siloxane (silicone) elastomers are a special group of organopolysiloxanes used on the skin. They are all characterized by different features, which demonstrate a positive therapeutic effect, e.g., on keloids, hypertrophic scars. In this case, the adhesive properties, ability to occlusion and diffusion gasses (oxygen, water vapor), which are presumably the factors of therapeutic action, were considered to be significant. The kind of elastomer, conformation of the chains and degree of its cross-linkage are important factors affecting its properties. One of such features is the ability to permeate of gasses (oxygen, water vapor), as well as the incorporation of medicinal substance and its release. The rate of diffusion is dependent on the size of the space formed between cross-linked chains and chains density (degree of cross-linking). These are so called free permeable channels. The larger the diameter of the channels in relation to the size of water vapor, oxygen, or active ingredient particles, or number of the channels, the easier and faster the diffusion process. The formation of free permeable channels is possible due to the high flexibility of Si-O bond, and thus flexibility of siloxane chains, as well as their mobility and ability to deform (De Buyl, 2001; Butts et al., 2002; Kurian et al., 2003; Zhang and Cloud, 2006; Lin et al., 2009; Czech et al., 2011).

**Table 2** presents the most important properties of siloxanes, which cause their common application as an active compound or auxiliary substance in medicinal products, medical devices, and cosmetics (Kuo, 1999; De Buyl, 2001; Clarson, 2003; Lin et al., 2009; Mojsiewicz-Pieńkowska, 2011, 2014, 2015; Tiwari and Soucek, 2014).

**Table 2:** The most important features of siloxanes used in medicinal products, medical devices and cosmetics.

No.	Siloxanes properties		
	**General**	**Conditioning medical and cosmetic application**	**Conditioning medical application on skin (elastomers)**
1	Low toxicity	No effect on the immune system	Biocompatibility
2	Stability-resistance to temperature (-100 to 250°C)	No carcinogenic effect	Highly non-toxic
3	Stability-resistance to atmospheric condition	Do not irritate skin or have low irritation	Ability to adhesion
4	Stability-aging resistance	Do not foster the growth of microorganisms	Anti-adhesion
5	Stability-resistance to oxidation	Safety of application due to the lack of penetration through cell membranes	Ability to permeation of water vapor and oxygen
6	Stability-resistance to moisture	Similar to hydrophilic/lipophilic properties of human skin	Ability to skin occlusion
7	Stability-resistance to UV radiation	Lack of reactivity with respect to the active pharmaceutical ingredient and excipients	Hydration of *stratum corneum*
8	Resistance to degradation	Similarity to biological membranes	Possibility of the diffusion of the active substance
9	Low chemical reactivity	Clearness, colorless, odorless	Transparency
10	Low surface energy	Easy spreading	Non-visible on the skin
11	Low surface tension	Low heat of vaporization	Resistance to extensibility and deformation
12	Smooth texture	Compatible with a wide range of solvents	Flexibility
13	Hydrophobicity	Imparts a soft-silky feel to the skin	
14	Water repellent		
15	Hydrophilicity		

## Summary

Generally, siloxanes (silicones) are well tolerated by the human organism, and therefore they are an integral part of innovative methods of treatment, health care and nursing. They are commonly regarded as non-toxic to humans and the environment, or toxic to a very small extend. However, there is a number of publications in which the scientists and experts question this opinion. Many authors demonstrated that the degree of polymerization and the structure affect the ability to overcome cellular barriers, including *stratum corneum* of the skin and absorption into the organism, migration in the living organism, ability to accumulate, degradability and toxicity. This particularly applies to low molecular weight siloxanes. It can be concluded in the summary, that an evaluation of the safety of siloxanes application should always refer to a particular compound, not a chemical group. Furthermore, the use of low molecular weight silicones should be reduced, as well as the purity of high molecular weight silicones, which may contain low molecular compounds as impurities, should be monitored. It should be emphasized that in the case of silicones for medical and pharmaceutical use, the manufactures of this group of compounds formed a special class, which they called “Medical Grade Silicones” or “Silicones for Healthcare Application.” These silicones must meet certain standards. Medical grade silicones are specially designed, produced and purified, so that to meet the highest requirements of the medical industry. The detailed toxicity data and information about “Medical Grade Silicones” and “Silicones for Healthcare Application” will be given in the next parts of this cycle.

## Author Contributions

KM-P: conception and design of work including the figures, collecting the literature, scientific description of the comments, corresponding author; MJ: preparation of the graphical figures, co-operation in the manuscript preparation; KS: co-operation in collecting the literature, co-operation in the manuscript preparation; DK: co-operation in collecting the literature, co-operation in the manuscript preparation. All authors critically revised the manuscript and approved the final version.

## Conflict of Interest Statement

The authors declare that the research was conducted in the absence of any commercial or financial relationships that could be construed as a potential conflict of interest.
